# Epidemiology of Dry Eye in Patients With Autoimmune Disease

**DOI:** 10.1001/jamanetworkopen.2025.60275

**Published:** 2026-02-23

**Authors:** Nan-Ni Chen, Yu-Tung Huang, Chi-Chin Sun

**Affiliations:** 1 Department of Ophthalmology, Chang Gung Memorial Hospital, Chiayi, Taiwan; 2 National Center for Geriatrics and Welfare Research, National Health Research Institutes, Yunlin, Taiwan; 3 Colleges of Medicine, School of Medicine, Chang Gung University, Taoyuan, Taiwan; 4 Department of Ophthalmology, Chang Gung Memorial Hospital, Keelung, Taiwan

## Abstract

This cohort study investigates the prevalence, onset, and ocular surface damage of dry eye disease across major autoimmune diseases to clarify disease-specific characteristics among patients in Taiwan.

## Introduction

Dry eye disease (DED) is a multifactorial disorder of the tear film and ocular surface affecting 5% to 40% of adults aged more than 40 years in Taiwan.^1^ Autoimmune diseases are major systemic factors, and DED represents their most common ocular manifestation, occurring in 10% to 95% of affected patients.^1,2^ Autoimmune-related DED encompasses both aqueous-deficient and evaporative components and generally shows more severe inflammation and symptoms than nonautoimmune DED.^1,3^ This study investigates the prevalence, onset, and ocular surface damage of DED across major autoimmune diseases to clarify disease-specific characteristics.

## Methods

This cohort study analyzed data from the Taiwan National Health Insurance Research Database (2008–2021), which covers more than 99% of residents. Ten autoimmune diseases were identified through the Registry for Catastrophic Illness Patients (eTable in Supplement 1). Definitions, statistical analysis, software, statistical significance information, and study dates are available in eMethods of Supplement 1. Ethical approval was granted by the Chang Gung Medical Foundation and the requirement for patient consent were waived owing to retrospective design and use of anonymized data. This study followed the STROBE reporting guideline. Statistical significance was set at a 2-sided *P* < .05.

## Results

A total of 67 264 patients newly diagnosed with autoimmune diseases from 2011 to 2020 were analyzed. The most prevalent diseases were rheumatoid arthritis (24595 [36.6%]) and Sjögren syndrome (23 088 [34.3%]). DED prevalence varied by diseases, highest in Sjögren syndrome (18 766 of 23 088 [81.3%]) and lowest in Crohn disease (309 of 1346 [23%]) and was consistently higher in females (Table 1). Keratitis and corneal ulcer occurred most frequently in Sjögren syndrome (keratitis, 7097 [30.7%]; corneal ulcer, 766 [3.3%]), followed by rheumatoid arthritis (keratitis, 5299 [21.6%]; corneal ulcer, 727 [3.0%]), systemic lupus erythematosus (keratitis, 2320 [23.8%]; corneal ulcer, 279 [2.9%]), and vasculitis (keratitis, 565 [27.1%]; corneal ulcer, 53 [2.5%]). Differences in the proportion of DED cases progressing to keratitis or ulcers were statistically significant, with vasculitis showing the highest progression (keratitis, 94 of 341 [27.6%]; ulcer, 36 of 341 [10.6%]).

**Table 1.** Prevalence of Dry Eye Disease Across 10 Autoimmune Diseases by Sex in Taiwan

Autoimmune disease	Total cases, No.	Dry eye disease, No. (%)		
		Total	Male	Female
Sjogren syndrome	23 088	18 766 (81.28)	1712 (68.23)	17 054 (83.03)
Rheumatoid arthritis	24 595	9655 (39.26)	1708 (27.26)	7947 (43.78)
SLE	9732	3711 (38.13)	401 (26.77)	3310 (40.94)
Polymyositis	611	221 (36.17)	48 (24.00)	173 (42.20)
Systemic sclerosis	1248	432 (34.62)	89 (23.86)	343 (39.49)
Pemphigus	959	300 (31.28)	120 (25.75)	180 (36.98)
Dermatopolymyositis	836	260 (31.10)	82 (27.15)	178 (33.21)
Ulcerative colitis	2764	821 (29.70)	413 (24.01)	408 (40.57)
Vasculitis	2085	575 (27.58)	223 (19.89)	352 (36.59)
Crohn disease	1346	309 (22.96)	163 (18.80)	146 (32.37)

Abbreviation: SLE, systemic lupus erythematosus.

DED typically preceded autoimmune disease by approximately 3 years (Table 2). Across all autoimmune conditions, patients with DED were diagnosed at significantly older ages than those without DED. The largest difference occurred in Crohn disease, where DED patients were diagnosed at a mean (SD) age of 53.57 (18.00) years compared with 39.57 (15.66) years for patients without DED (*t* = –10.51; *P* < .001), underscoring a temporal and clinical association between DED and autoimmune disease onset.

**Table 2.** Age at Autoimmune Disease Diagnosis in Patients With and Without Dry Eye Disease

Autoimmune disease	Dry eye, mean (SD)		Without dry eye, diagnosis age, mean (SD), y	*t* Value	*P* value
	Age difference, y^a^	Diagnosis age, y			
Sjogren syndrome	−3.50 (3.72)	57.81 (13.10)	53.68 (14.98)	−20.21^b^	<.001
Rheumatoid arthritis	−2.85 (4.39)	58.85 (13.23)	53.60 (14.12)	−27.79^b^	<.001
SLE	−2.75 (4.20)	48.15 (17.17)	41.59 (16.74)	−16.54	<.001
Polymyositis	−2.95 (4.67)	55.81 (15.06)	51.13 (14.32)	−3.53	<.001
Systemic sclerosis	−3.01 (4.19)	58.93 (12.61)	53.67 (13.84)	−6.4^b^	<.001
Pemphigus	−2.96 (4.38)	58.78 (14.77)	53.60 (15.81)	−4.31	<.001
Dermatopolymyositis	−3.05 (4.33)	57.43 (14.13)	51.99 (13.58)	−4.81	<.001
Ulcerative colitis	−3.62 (4.67)	52.09 (14.73)	45.56 (14.99)	−9.16	<.001
Vasculitis	−2.65 (4.44)	47.51 (15.68)	43.28 (15.65)	−4.11	<.001
Crohn disease	−3.72 (4.57)	53.57 (18.00)	39.57 (15.66)	−10.51^b^	<.001

Abbreviation: SLE, systemic lupus erythematosus.

^a^ Age difference between diagnosis of autoimmune diseases and dry eye.

^b^ Unequal variances *t.*

## Discussion

DED prevalence was highest in Sjögren syndrome. DED often preceded autoimmune disease diagnosis by approximately 3 years, suggesting that ocular surface involvement may represent an early systemic manifestation and provide an opportunity for earlier clinical evaluation, despite possible diagnostic delay. Patients with DED were generally older at autoimmune disease onset. Similar observations in systemic lupus erythematosus with secondary Sjögren syndrome have been reported,^4^ potentially reflecting hormonal, genetic, or environmental factors, or a distinct disease subset, while findings in Crohn disease may be associated with its bimodal age distribution.^5^ Progression to corneal ulcer was more frequent in vasculitis, rheumatoid arthritis, and systemic lupus erythematosus, indicating more severe ocular inflammation, likely driven by shared genetic and immune pathways.^6^

This study is limited by the lack of detailed ophthalmic examinations, symptom severity, biomarker, and treatment data, precluding assessment of disease activity and treatment effects. Disease severity definitions based on keratitis or corneal ulcer may have included non-DED etiologies. Nonetheless, these findings highlight the importance of early recognition and proactive management of DED to reduce ocular morbidity in autoimmune populations.

## Notes

Supplement 1. **eTable.** List of ICD-9-CM and ICD-10 codes to define the ten types of autoimmune disorders
eMethods

Supplement 2.
Data Sharing Statement
