# Supplementary material for: Epidemiology of Dry Eye in Patients With Autoimmune Disease
Source: JAMA Netw Open. 2026 Feb 23;9(2):e2560275. doi: 10.1001/jamanetworkopen.2025.60275 (PMC12931465; doi:10.1001/jamanetworkopen.2025.60275)
Supplement: Supplement 1. — eTable. List of ICD-9-CM and ICD-10 codes to define the ten types of autoimmune disorders eMethods [file jamanetwopen-e2560275-s001.pdf]

Supplemental Online Content

Chen N, Huang Y, Sun C. Epidemiology of dry eye in patients with autoimmune disease. *JAMA Netw Open.* 2026;9(2):e2560275.
doi:10.1001/jamanetworkopen.2025.60275

eTable. List of ICD-9-CM and ICD-10 codes to define the ten types of autoimmune disorders

eMethods.

This supplemental material has been provided by the authors to give readers additional information about their work.

© 2026 Chen N et al. *JAMA Network Open*.

eTable. List of ICD-9-CM and ICD-10 codes to define the ten types of autoimmune disorders

Autoimmune disease	ICD-9-CM	ICD-10-CM
Sjogren syndrome	710.2	M35.00-M35.09
Rheumatoid Arthritis	714.0, 714.30-714.33	M05.70-M06.09, M06.20-M06.39, M06.80-M06.89, M06.9, M08.00-M08.99,
SLE	710.0	M32.0-M32.9
Polymyositis	710.4	M33.20-M33.29
Systemic Sclerosis	710.1	M34.0-M34.9
Pemphigus	694.4	L10.0-L10.9
Dermatopolymyositis	710.3	M33.00-M33.19, M33.90-M33.99, M36.0
Ulcerative colitis	556.0-556.6, 556.8-556.9	K51.00-K51.919
Vasculitis	136.1, 443.1, 446.0, 446.1, 446.2, 446.4, 446.5, 446.7	I73.1, M30.0, M30.2, M30.8, M31.0, M31.30, M31.31, M31.4, M31.5, M31.6, M35.2
Crohn's disease	555	K50.00-K50.919

Note: SLE, Systemic Lupus Erythematosus

© 2026 Chen N et al. *JAMA Network Open*.

eMethods.

Dry eye disease (DED) was defined as at least two ophthalmology outpatient visits with a diagnosis defined by diagnostic codes for dry eye syndrome, keratoconjunctivitis sicca, or sicca syndrome. Severity of ocular surface damage was further categorized as superficial punctate keratitis or corneal ulcer. The index date corresponded to the certification date of each autoimmune disease. Prevalence and onset age differences between autoimmune diseases and DED were assessed using t tests and ANOVA (SAS 9.4). Data were analyzed from November 13, 2023, through March 1, 2024.

© 2026 Chen N et al. *JAMA Network Open*.
