# Supplementary material for: Epidemiology of Dry Eye in Patients With Autoimmune Disease
Source: JAMA Netw Open. 2026 Feb 23;9(2):e2560275. doi: 10.1001/jamanetworkopen.2025.60275 (PMC12931465; doi:10.1001/jamanetworkopen.2025.60275)
Supplement: Supplement 2. — Data Sharing Statement [file jamanetwopen-e2560275-s002.pdf]

Data Sharing Statement

Chen. Epidemiology of Dry Eye in Patients With Autoimmune Disease. *JAMA Netw Open*. Published February 23, 2026. doi:10.1001/jamanetworkopen.2025.60275

Data
Data available: No
